# Buccal Spectral Markers for Lung Cancer Risk Stratification

**DOI:** 10.1371/journal.pone.0110157

**Published:** 2014-10-09

**Authors:** Andrew J. Radosevich, Nikhil N. Mutyal, Jeremy D. Rogers, Bradley Gould, Thomas A. Hensing, Daniel Ray, Vadim Backman, Hemant K. Roy

**Affiliations:** 1 Biomedical Engineering Department, Northwestern University, Evanston, Illinois, United States of America; 2 Biomedical Engineering Department, University of Wisconsin, Madison, Wisconsin, United States of America; 3 Department of Medicine, NorthShore University HealthSystems, Evanston, Illinois, United States of America; 4 Department of Medicine, Boston University, Boston, Massachusetts, United States of America; Universität Bochum, Germany

## Abstract

Lung cancer remains the leading cause of cancer deaths in the US with >150,000 deaths per year. In order to more effectively reduce lung cancer mortality, more sophisticated screening paradigms are needed. Previously, our group demonstrated the use of low-coherence enhanced backscattering (LEBS) spectroscopy to detect and quantify the micro/nano-architectural correlates of colorectal and pancreatic field carcinogenesis. In the lung, the buccal (cheek) mucosa has been suggested as an excellent surrogate site in the “field of injury”. We, therefore, wanted to assess whether LEBS could similarly sense the presence of lung. To this end, we applied a fiber-optic LEBS probe to a dataset of 27 smokers without diagnosed lung cancer (controls) and 46 with lung cancer (cases), which was divided into a training and a blinded validation set (32 and 41 subjects, respectively). LEBS readings of the buccal mucosa were taken from the oral cavity applying gentle contact. The diagnostic LEBS marker was notably altered in patients harboring lung cancer compared to smoking controls. The prediction rule developed on training set data provided excellent diagnostics with 94% sensitivity, 80% specificity, and 95% accuracy. Applying the same threshold to the blinded validation set yielded 79% sensitivity and 83% specificity. These results were not confounded by patient demographics or impacted by cancer type or location. Moreover, the prediction rule was robust across all stages of cancer including stage I. We envision the use of LEBS as the first part of a two-step paradigm shift in lung cancer screening in which patients with high LEBS risk markers are funnelled into more invasive screening for confirmation.

## Introduction

Lung cancer remains the leading cause of cancer deaths in the US with over 150,000 deaths per year (86,930 males and 72,330 females) [1]. While localized lung cancer is curable through surgical resection, the insidious nature of the disease means that its symptoms only become apparent in the advanced and hence incurable stages. This is a major factor behind the dismal 16% 5 year survival rate of patients diagnosed with lung cancer, underscoring the need for screening to identify the pre-symptomatic population. Given that the at-risk population is easily identifiable (current/former smokers constitute ∼90% of lung cancer patients), it should be feasible to develop a pre-screening test that assesses their risk of developing cancer. However, this risk group is dauntingly large, encompassing approximately one quarter of the entire adult population.

Previous attempts to screen the at-risk population have been plagued by lack of sensitivity for lung cancer (i.e., chest X-ray or sputum cytology). Recently, there has been considerable excitement with the much more sensitive low dose computed tomography scans (LDCT). Indeed, in the landmark National Lung Cancer Screening Trial (NLST) of 53,439 patients there was ∼20% reduction in lung cancer mortality for the LDCT group compared to chest X-ray. This served as the impetus for a variety of groups (including the US Preventive Services Task force) to recommend screening high risk populations. Unfortunately, while the sensitivity of LDCT was excellent (93.8%) the specificity was only 73.4% compared to chest X-ray with specificity of 91.3% [2]. The problems associated with this modest specificity are accentuated by the low prevalence of lung cancer (1.1%) despite the use of very stringent inclusion criteria (age 55–74, ≥30-pack-years or quit smoking within the last 15 years). Thus, the key metric in a screening test, the positive predictive value (PPV) was a dismal 3.8%. In other words, the vast majority of positive tests were in fact false positives. Moreover, 27.3% of first round tests had some positive results, obligating further testing (radiographic or invasive procedures) with all the incumbent costs, patient worry and potential complications. In order to mitigate the harms associated with a low PPV, it is critical to enrich the population undergoing LDCT with those patients who are most likely to harbor lung cancer.

Our multi-disciplinary group focuses on bridging the gap between biomedical optics and cancer screening risk stratification. Our approach has centered on identifying at-risk patients through application of field carcinogenesis (also known as field of injury, field effect, field defect, etc.). Field carcinogenesis is the well-established proposition that the genetic/environmental milieu that leads to neoplastic transformation should be detectable diffusely throughout the field of injury. Focal tumors can then emerge from the field through final stochastic events (i.e., mutations in the critical tumor suppressor gene/proto-oncogene). The corollary of these diffuse genetic/epigenetic events is structural alterations occurring on the micro and nano-scale (e.g., high order chromatin compaction, cytoskeletal alterations, extracellular matrix reorganization, etc). In order to detect such micro/nano-scale alterations we invented a novel technology, low coherence enhanced backscattering technology (LEBS) that specifically targets changes in structures between 20 nm and 3 microns in size [3]–[5]. Given that many of these changes are larger than the diffraction limit of light, the mucosa appears histologically normal. Thus, LEBS can detect the intracellular (i.e., cytoskeleton, ribosomes, mitochondria, and nucleus) and extracellular (i.e., collagen matrix cross-linking) alterations known to altered in early carcinogenesis even in the histologically normal mucosa.

Using a first generation bench-top system, we have shown that the LEBS signatures were able to detect the presence of colorectal and pancreatic field carcinogenesis through analysis of the rectal and duodenal mucosa, respectively [6], [7]. Indeed, in a study of 297 patients, biopsies of the endoscopically normal rectum were able to predict significant neoplasia throughout the colon with diagnostic performance (sensitivity 100%, specificity 80% for advanced adenomas) [7]. Moreover, we showed that the origin of the changes in colon field carcinogenesis was primarily in alterations of structures smaller than ∼200 nm found within the top 600 microns of rectal mucosa [3].

In order to apply this approach to lung cancer, we targeted the buccal (cheek) mucosa since it is well-established as a part of the field of injury from cigarette smoking. In fact, the cheek epithelium has been referred to as “molecular mirror” or “window to the soul” of lung carcinogenesis [8]–[10]. In this study, we wanted to assess whether buccal LEBS markers could discriminate between smokers with and without lung cancer. To accomplish this goal, we employed a newly developed LEBS fiber optic probe to allow in situ, painless interrogation of the buccal mucosal.

## Materials and Methods

### Participants

**Figure 1:**
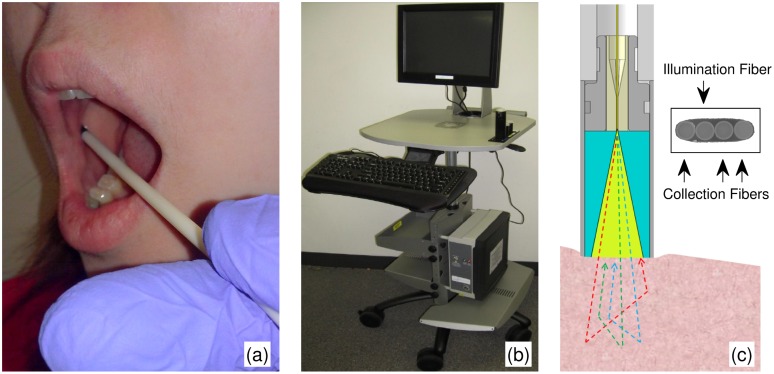
Clinical LEBS instrument. (a) LEBS probed inserted into the cheek for measurement of the buccal mucosa. (b) Portable cart that houses the data acquisition instrumentation and software. (c) 3.4 mm diameter fiber-optic LEBS probe schematic. White light from one illumination fiber is directed onto the sample. Three collection fibers detect the scattered light intensity as a function of angle and illumination wavelength. The inset shows an image of the linear optical fiber array.

This case-control study was approved by the Institutional Review Board at NorthShore University HealthSystem. All participants provided written informed consent prior to enrollment in the study. Cases had pathologically confirmed primary lung cancer without previous chemotherapy or radiation. Controls were smokers (current or past) without a diagnosis or symptoms of lung cancer. LEBS readings of the buccal mucosa were taken from oral cavity by a 3.4 mm diameter fiber optic LEBS probe with gentle contact (∼10 locations assessed, each requiring 250 milliseconds). Figure 1a shows the LEBS probe inserted into the cheek for measurement of the buccal mucosa and Figure 1b shows the portable cart housing the data acquisition module. LEBS measurements were acquired by trained technicians, while the data analysis was performed by the investigators. Both were blinded to the pathology findings at the time of data acquisition and analysis. The investigators became un-blinded only to perform statistical analysis.

### LEBS Analysis of tissue micro/nano-structure

The characterization of tissue micro/nano-structure in field carcinogenesis has been detailed elsewhere [3], [11]. We therefore review only the basic principles needed in this paper. The primary physical characteristic that describes both tissue micro/nano-structure as well as the light scattering detected by LEBS is the spatial auto-correlation function *B*(*r*) [12], [13]. *B*(*r*) is a statistical quantity that quantifies the range of structures that make up a specific tissue sample. The shape of *B*(*r*) can be described by three physical parameters. 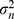 is the fluctuation strength of spatial tissue heterogeneity, 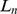 is the characteristic structural length-scale, and 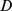 is the shape of the spatial distribution. By using scattering theory and simple mathematical transformations [14], these three physical properties can be related to the optical scattering properties 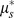 (the reduced scattering coefficient) and 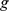 (the anisotropy factor).

The LEBS phenomenon is a coherent intensity peak that forms due to the tissue heterogeneity specified by *B*(*r*). By using illumination with partial spatial coherence, LEBS is able to target the short light scattering paths that retain information about 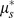, 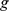, and 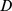 [15]–[18]. To quantify the shape of the LEBS peak, we use three empirical parameters: enhancement (E, the height of the peak), full width at half maximum (W), and spectral slope (S, change in E per unit wavelength). These three empirical parameters encode information that allows us to calculate the optical and physical properties of biological tissue.

### LEBS fiber optic probe instrumentation and markers

The design and theory behind the fiber-optic LEBS probe (assembled by OFS, Avon, CT) have been detailed elsewhere [4], [19]. Briefly, the LEBS probe consists of a linear array of 4 optical fibers shown in Figure 1c. The first fiber provides white light illumination onto the tissue surface, and the remaining three fibers collect light at three backscattering angles (−0.6°, 0.6° & 1.12° relative to the incident direction). Light traveling through the three collection fibers is detected by a spectrometer optimized for wavelengths between 500 and 700 nm. The LEBS probe therefore measures the backscattered light signal as a function of wavelength and angle. Finally, in order to control the illumination spatial coherence length (L_SC_) (and thereby limit the light penetration depth), a 9 mm glass rod spacer separates the optical fibers from the tissue surface. Buccal mucosa consists of a thick layer (∼500–800 µm) of non-keratinized stratified squamous epithelium. In order to target the squamous cells within the top ∼100–150 µm of mucosa that interact most closely with the oral environment, we restricted the spatial coherence length to 27 µm at 700 nm illumination wavelength [4], [20].

In order to characterize the LEBS signal, two empirical parameters are calculated: the enhancement at θ = 0.6° (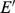) and the normalized spectral slope (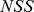). These parameters were chosen in part due to their past sensitivity to colorectal and pancreatic field carcinogenesis [6], [7]. To calculate these parameters we first calculate the spectrally resolved 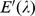 by taking the average of the intensities at −0.6° and +0.6° minus the intensity at 1.12°. The 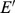 parameter is then simply the average of 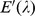 from 610 to 690 nm. Next we calculate the spectral slope (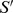) of 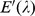 using a linear regression of the form 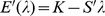 over the wavelength range 610–690 nm. Since the 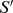 parameter is partially correlated with 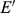, we must remove this dependence in order to calculate an independent marker. We therefore calculate 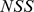 by dividing 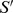 by 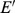 and multiplying by the average wavelength: 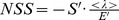.

To gain a more physical understanding of the tissue composition, we also derive the physical/optical properties 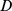 and 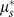 using the empirical parameters 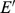 and 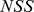. To do this, we begin with the assumption that 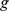 = 0.9 in biological tissue [21]. We then use empirical equations formulated using Monte Carlo simulation to calculate 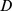 and 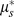 [22]: 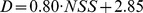 and 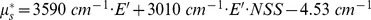. Prior to applying these equations, the value of 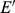 was first multiplied by a constant calibration factor of 0.31 to achieve agreement between experiment and Monte Carlo simulation. Possible reasons for the difference in value between theory and experiment are discussed in Ref. [17].

A number of data exclusion criteria were employed to ensure the robustness of the dataset. First, we removed patients whose values for either 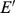 or 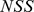 were outside of the range [Q_1_–1.5 (Q_3_−Q_1_), Q_3_+1.5 (Q_3_−Q_1_)], where Q_1_ and Q_3_ are the 25^th^ and 75^th^ percentiles, respectively. For normally distributed data this corresponds to ±2.7 standard deviations. Furthermore, we excluded values of 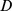 and 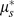 that were determined to be unphysical for buccal mucosa. These criterion were 1<
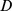
<4 and 0 cm^−1^<
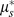
<100 cm^−1^.

### Statistical analysis

The empirical parameters 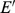 and 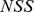 were combined into a single diagnostic biomarker (termed the LEBS marker) to predict lung cancer using a multivariable logistic regression performed in MATLAB R2013a. The final prediction rule is generated as a linear combination of 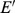 and 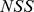:
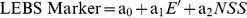
(1)where a_n_ are coefficients assigned by the MATLAB function ‘mnrfit’. To characterize the overall diagnostic performance we calculated the sensitivity, specificity, and the accuracy by generating the receiver operating characteristic (ROC) curve using the MATLAB function ‘perfcurve’. Contributions of confounding factors (age, race, smoking/alcohol status, and personal and family history of cancer) toward the LEBS marker were evaluated by performing analysis of covariance (ANCOVA) in STATA 8.0.

To test the robustness of the LEBS marker we chronologically separated our dataset into a training and validation set. The coefficients for the prediction rule in Eq. 1 as well as the optimal test threshold value were developed using patients in the training set. These values were then applied to the blinded validation set.

## Results

### Patient Characteristics

Demographics for our study are shown in Table 1. The total dataset consisted of 79 patients, of which 6 non-smoking controls were not included in the prediction rule. The remaining dataset of 73 patients comprised of 27 smoking controls without lung cancer and 46 lung cancer patients with a smoking history. These patients were chronologically divided into two similarly-sized datasets: the first 32 patients were included in the training sets, and the remaining 47 patients (including all 6 non-smoking controls) were assigned to the blinded validation set.

**Table 1:** Patient Demographics.

Patient Type	Age (mean±SD)	Gender (% Male)	Race (% Caucasian)	# Patients (Training)	# Patients (Validation)
Non-Smoking Controls	57±6.83	50	83	0	6
Smoking Controls	59±11	52	74	15	12
Lung Cancer	70±11	48	87	17	29
All Patients	65±12	49	82	32	47

### Nature of the optical alterations occurring in lung cancer field carcinogenesis

**Figure 2:**
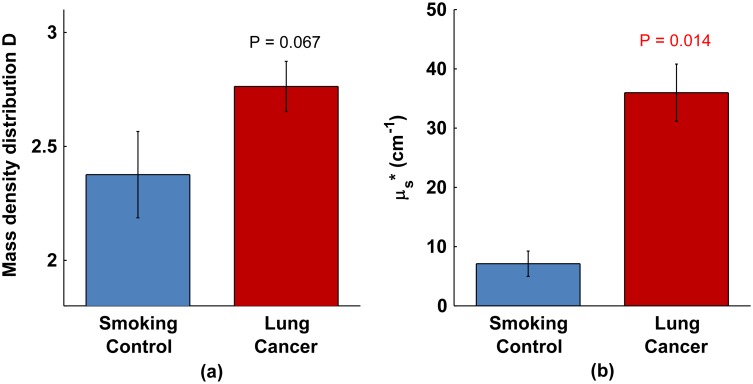
Optical properties are altered in buccal mucosa of patients with lung cancer compared to smoking controls. (a) Mass density distribution 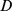. (b) Reduced scattering coefficient 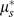.

Figure 2 shows the alterations in optical properties 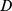 and 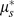 for the full dataset. For 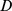 we measured a nearly significant 16% increase in value (P = 0.067). This increase indicates a shift in nano-structural composition towards larger sizes in lung field carcinogenesis. For 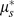 there was a significant 5 times increase in value (P = 0.014). This change is due in part to an increase in the variance of spatial mass density organization.

### Evaluation of the LEBS Marker

**Figure 3:**
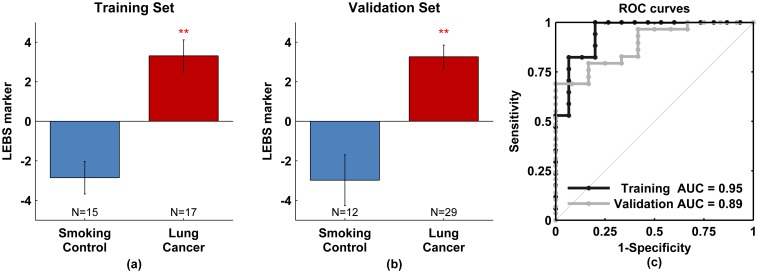
LEBS marker diagnostic power. Composite LEBS marker calculated using linear combination of LEBS parameters of 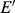 and 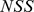 shows highly significant alteration (p<0.001) between smoking controls and lung cancer patients in both training and validation sets (panels a and b). c) Receiver operating characteristic (ROC) curve for training and validations sets. The area under the curve (AUC) is 0.95 for the training set and 0.89 for the validation set. The double red star indicates statistical significance at the 0.1% level.

The composite LEBS marker was evaluated as a linear combination of the empirical parameters 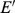 and 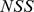 using data in the training set (Eq.1). This prediction rule was then applied to the validation set. The values of the LEBS marker calculated for the training and validation sets are shown in Figure 3a and 3b, respectively. In both the training and validation sets, the LEBS marker served as a highly sensitive predictor of the presence of lung cancer with P<0.001 in each case. Moreover, the absolute values of the LEBS marker were consistent across the training and validation sets with P = 0.93 between smoking control groups and P = 0.96 between lung cancer groups.

Summarizing the diagnostic potential of the LEBS marker, the receiver operating characteristic (ROC) curve for the training and validation sets are shown in Figure 3c. The performance in the training set was excellent with 94% sensitivity, 80% specificity, and 95% overall accuracy (95% confidence interval from 87% to 100%). Applying the same prediction rule to the validation set, we achieved 79% sensitivity, 83% specificity, and 89% overall accuracy (95% confidence interval from 79% to 99%). These test performance characteristics are summarized in Table 2. The slight decrease in the performance for the validation set may be attributable in part to the modest number of patients in each data set. Supporting this claim, we note that the accuracy of each data set falls within the 95% confidence interval of the other data set.

**Table 2:** Test performance characteristics for LEBS marker threshold 0.04.

Study Set	Sensitivity	Specificity	AUC [95% CI]
Training	94%	80%	95% [87,100]
Validation	79%	83%	89% [79,99]

### Influence of cancer type, stage, and location

We next wanted to assess whether buccal LEBS markers performance would be affected by cancer type, stage, and location. To this end, we combined the training and validation sets for further analysis.

**Figure 4:**
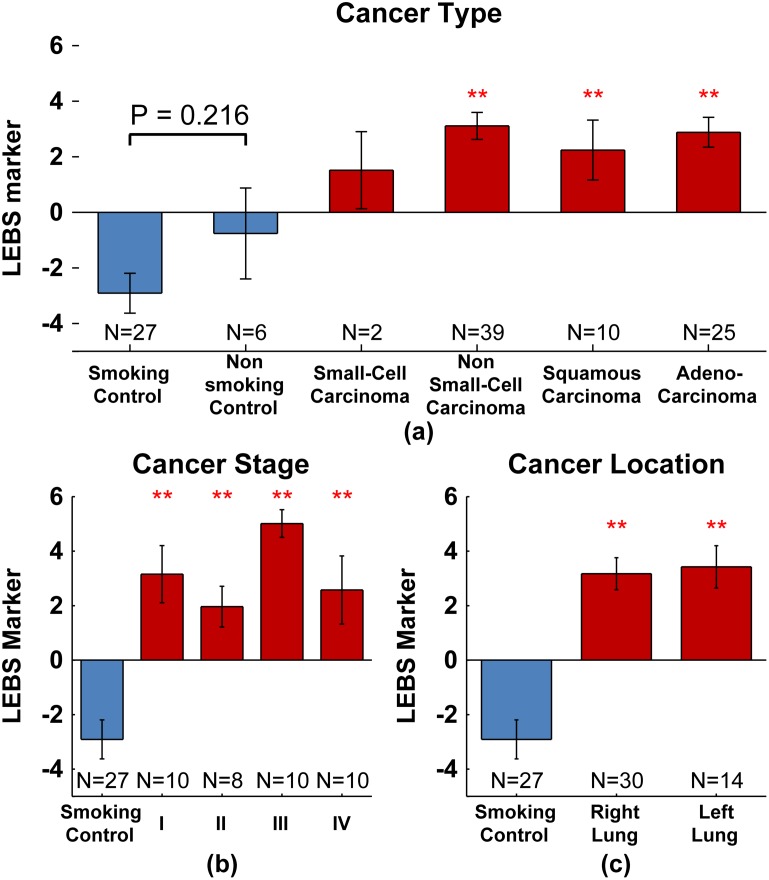
LEBS marker by cancer type, stage, and location. Composite LEBS marker separated according to cancer type (panel a), stage (panel b), and location (panel c). The double red star indicates statistical significance at the 0.1% level. In each panel, a number of cancer patients were not added to the subgroups due to incomplete pathology reports.

Figure 4a shows the LEBS marker values for lung cancer patients separated according to cancer type and control patients separated according to smoking status. We begin by noting that smoking and non-smoking controls are statistically indistinguishable with P = 0.216, suggesting that smoking status does not change the buccal morphology measured by LEBS. Next, we divided the cancer patients into small cell (N = 2) and non-small cell lesions (N = 39) with 5 patients remaining unclassified due to unknown histology. For small cell carcinomas we saw an increase in the LEBS marker relative to smoking controls (indicating increased aggressiveness), but due to limited patient numbers this change was insignificant (P = 0.112). For the non-small cell lesions that comprised a majority of the cancer patients, there was a highly significant (P<0.001) increase in the value of the LEBS marker. Finally, we further separated the non-small cell carcinoma group into squamous carcinomas (N = 10) and adenocarcinomas (N = 25) with 4 patients cancer type unknown. For both squamous carcinomas and adenocarcinomas there was a highly significant increase in the LEBS marker (P<0.001).

Figure 4b separates the lung cancer patient according to their stage: Stage I N = 10, Stage II N = 8, Stage III N = 10 and Stage IV N = 10 (8 patients cancer stage was unclassified). Regardless of cancer stage, the LEBS marker is a highly significant predictor of presence of lung cancer with P≤0.001. Similarly, Figure 4c shows that the LEBS marker is equally sensitive to lesions found in both the right and left lung (P<0.001 in each case).

### Potential Confounders

To study the effect of confounding factors on our results, we performed an analysis of covariance (ANCOVA) with the LEBS marker as the dependent variable and the presence of neoplasia, smoking (pack-years) history, alcohol history, race, gender, age, and personal and family history of cancer as predictors (Table 3). After incorporating these confounding factors into our model, the LEBS marker remained a highly significant predictor for the presence of lung cancer with P<0.001. At the same time, each of the confounding factors had an insignificant effect (P>0.05) on the LEBS marker.

**Table 3:** Confounding Factor Analysis.

Factor	ANCOVA P-value
Lung Cancer	<0.001
Age	0.80
Race	0.72
Gender	0.07
Smoking History (Pack-Years)	0.72
Alcohol Use	0.10
Personal History	0.75
Family History	0.57

## Discussion

We demonstrated herein that buccal interrogation with LEBS was able to discriminate between smokers with and without concurrent lung cancer. Importantly, buccal LEBS was able to sense both early and late stage lung cancer. Furthermore, while the dataset was modest, there did not appear to be any significant differences based on tumor histology. From a diagnostic point of view, the performance seemed excellent with only two optical markers, thereby avoiding concerns of over fitting. Furthermore, use of an independent validation set which is critical for any biomarker development, underscored the potential robustness of the findings.

From a clinical perspective, LEBS can help address the inefficiencies in the current screening approaches for lung cancer which result in most positive test results being false positives (∼99%). Indeed, since the prevalence of the disease is low even in the high risk population (90% of smokers will never get lung cancer), most positive results will turn out to be false positive. Therefore, using a test that enables a subset of patients to eschew more invasive procedures (e.g., LDCT) will enrich the screening population with those patients at highest risk for lung cancer, and therefore decrease the total number of false positives. For instance, Kovalchik and colleagues risk stratified patients using a relatively cumbersome clinical index, showing an improvement in the number of patients needed to be screened in order to prevent one lung cancer death for higher risk patients (302 in the entire NLST cohort versus 171 and 161 in the top two risk quintiles, respectively) [23]. However, the top two quintiles only encompassed approximately two thirds of cancer cases, thus underscoring the tradeoff between minimizing false positives and maximizing patients. This clinical concern is underscored by the fact that when the NLST criterion was applied to the Prostate, Lung, Colorectal, and Ovarian (PLCO) Cancer Screening Trial cohort, only 72% of lung cancer patients would have been eligible for LDCT [24].

To improve the effectiveness of cancer screening, a two-step approach has frequently been advocated. Under this approach the patient’s risk of disease is first assessed using a minimally invasive risk stratification technique. Patients found to be at high risk are then funneled into more invasive procedures for confirmation. For example, in colorectal cancer a fecal test followed by colonoscopy or in cervical cancer a Pap smear to decide which patients need colposcopy. While there are a number of promising risk stratification techniques being developed (including serum and sputum tests), these by and large rely on detecting minute amounts of circulating or expectorate tumor products which can be quite challenging. Therefore, evaluating field carcinogenesis is particularly attractive since it reflects the complex interactions between a patient’s genetics and the environmental insult (smoking, air quality, etc). Such interactions may determine why only a small fraction of smokers ever develop lung cancer.

Lung cancer epitomizes the field effect of carcinogenesis. This concept posits that the shared genotoxic milieu (both environment/smoking and genetic) that results in a focal neoplastic lesion in the lung also diffusely fosters an increased mutational rate (“fertile field”), which then stochastically leads to tumors. Indeed, the entire aero-digestive mucosa is “condemned” by tobacco exposure and thus patients with lung cancer are also at risk for oro-pharyngeal, esophageal squamous and second primary lung cancers. This is well established in the bronchial mucosa with changes in EGFR, p53, gene methylation, etc having previously been observed [25]. Additionally, there have been numerous gene expression studies on smoking-induced alterations in the bronchial transcriptosome [26], [27]. Importantly, while some of these changes reversed with smoking cessation, others were irreversible consonant with the long term risk in former smokers.

While the concept of field carcinogenesis has long been established, it is only recently that it has been exploited for diagnostics. For instance, in a landmark report by Spira and colleagues, they noted a panel of 80 genes obtained from the normal right mainstem bronchus had 80% sensitivity and 84% specificity for discriminating between the controls and cancers [8]. More recently, Blomquist and colleagues used a 14 gene set from bronchial mucosa and achieved an AUC of 0.82–0.87 for discriminating control smokers from those with cancer [28]. Thus, previous studies have demonstrated the potential clinical relevance of assaying field carcinogenesis.

From a teleological perspective, field carcinogenesis assessment represents an attractive approach for lung risk analysis. Specifically, while cigarette smoking is responsible for the vast majority of lung cancer, only a small proportion of smokers (∼10%) will ever develop lung cancer. While part of this is determined by smoking factors (intensity, duration), this is only part of the issue since host factors are critical in determining neoplastic responsiveness to the carcinogenic insult of cigarette smoke. Factors such as family history, gender, polymorphisms in genes (especially those high-prevalence, low-penetrance genetic polymorphisms in metabolizing carcinogen), and markers of tissue damage (COPD etc) are well established to be correlated as modifiers of environmental lung cancer risk. Field carcinogenesis is particularly attractive since it reflects the complex interactions between environmental risk factors and host susceptibility.

For techniques using field carcinogenesis to be feasible for population-wide screening, a less intrusive approach is required. In this regard, it has been noted that smoking-induced alterations in bronchial epithelial transcriptosome could also be detected in the buccal mucosa albeit this was somewhat obfuscated by salivary RNases [27]. Additionally, analysis of the buccal mucosa for glutathione S-transferase P1 expression, p16 methylation or allelic loss (9p21, 17p13 and 5q21) discriminated between smokers with and without concomitant lung cancer [29]. These findings have been mirrored from an ultra-structural perspective with buccal epithelial nuclear texture (DNA-specific Feulgen-thionin stain scanned with a high-resolution cytometer) or cellular organization with partial wave spectroscopic microscopy [30].

While our buccal LEBS data is biologically predicated upon the results of the techniques discussed above, the use of optics has some important potential advantages. First, the approach is particularly powerful because of its ease-of-use and convenience, especially with the minimally invasive 3.4 mm fiber optic LEBS probe. Furthermore, there may be particular advantages with regards to looking at tissue micro/nano-architecture. For instance, in essentially all cancer types that our group has studied (colon [3], [7], [11], pancreas [3], [11], and now lung) we have seen an increase in the mass density distribution 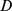. The fact that this tissue property changes in the same direction across organs in field carcinogenesis despite having quite different genetic/epigenetic makeup may represent a common pathway in early cancer progression. Indeed, the fact that a similar magnitude of the LEBS marker was seen regardless of lung cancer histology despite having different precursor lesions supports this idea (although we acknowledge that this is very speculative at this point).

While the biological underpinnings of field carcinogenesis remain incompletely understood, the optical parameter 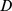 provides fundamental insights into tissue micro-structure. A complete discussion of the link between optical properties and tissue micro/nano-structure is beyond the scope of this clinical report, but has been detailed previously [3], [12]. Briefly, the increase in the value of 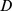 indicates a shift in tissue structure towards larger sizes. Examples of processes which could lead to such a change include chromatin condensation in the nucleus or collagen fiber cross-linking in the extracellular matrix. In fact, it bears mention that many of the physical alterations associated with field carcinogenesis are also recognized hallmarks of neoplastic transformation. For instance, when its value is smaller than 3, 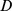 represents the mass fractal dimension, a physical parameter that is well established for cancer diagnosis and prognostication [31].

There are some limitations of this study that should be acknowledged. First, the modest size of this case-control study cannot provide a definitive determination of the diagnostic performance but rather demonstrates a first proof-of-principle. Importantly, the use of an independent dataset and only two LEBS markers supports the robustness of these findings. Second, this study focused exclusively on smokers and thus does not provide insight into whether this approach may be useful in the subgroup of lung cancer patients without smoking history (∼10% of all cases). Finally, the biological determinants of the micro/nano-structural alterations which the LEBS marker senses are incompletely elucidated. Evidence in colon suggests contributions from alterations in high order chromatin structure, cytoskeletal, and the extracellular matrix [11], [32], [33]. In addition, since buccal epithelium is heterogeneous the contribution of the neoplastic signals from various layers is unclear, suggesting that further optimization may lead to improved diagnostics.

In conclusion, we provide the proof-of-concept that buccal LEBS could discriminate between smokers with and without lung cancer. Importantly, this was validated on an independent dataset. The accuracy suggests clinical utility as does the non-invasive nature of the test (oral insertion of fiber optic probe). While larger multi-center studies are being initiated, if confirmed buccal LEBS may serve as pre-screen to funnel the most appropriate patients into diagnostic modalities such as LDCT. This would mitigate many of the potential harms of lung cancer screening that are related to the high false positive rate.

## Supporting Information

Data S1 **Biomarkers and patient characteristics used for diagnostic and confounding factor analysis.**
(XLSX)
